# Materia Medica Notes

**Published:** 1895-05

**Authors:** 


### MATERIA MEDICA NOTES.

One does not often think of Iodine as a remedy for diarrhœa, yet it is indicated for purulent stools, with cutting pains in the intestines, accompanied by nausea and vomiting and sour taste in the mouth. Several cases of winter diarrhœa have presented just such symptoms.

Iodine produces extreme emaciation and increases the appetite.

Iodine resembles Spongia in its therapeutic action. In children with black eyes Iodine is preferable; in children with blue eyes Spongia. The Iodine cough is moist but harsh; the

Spongia, dry, barking, rough, with suffocation spells. Spongia is useful (after Aconite) at the beginning of croupous inflammation. Iodine when the membrane is extensive, with jerking breathing.

In scrofulous patients, with dry coryza becoming fluent in the open air Iodine is beneficial; also when there is a chronic fetid discharge.

This is the season for neuralgias, and a few hints may be acceptable.

Stannum—When the pain increases gradually to its highest pitch and then gradually declines.

Mezereum—Periodical attacks of left-sided supra-orbital neuralgia, commencing in the morning, increasing until noon and then gradually subsiding until four P. M. (similar to Caust.).

Belladonna—Pains come on suddenly and as suddenly disappear. Face, red, hot, and bloated: aggravation from noise, light, motion, and touch.

Arsenicum—Periodical neuralgia, with burning, stinging pains, aggravated at night.

Amelioration from warm applications. Always to be thought of after the abuse of Quinine.

Spigelia—Ciliary neuralgia, worse about two A. M. Sharp, shooting pains radiate from the eye in every direction, and especially back into the head.

Rhus-tox—For facial or other neuralgia occasioned by getting wet. Stinging, burning, darting, and tearing pains, somewhat relieved by cold applications. Patient is very restless and feels somewhat better from moving about.

Causticum—Intermittent neuralgia coming on at nine A. M. and going off at three or four P. M. Hardness of the muscles in the region of the pain. The pains are tearing in character.

Chelidon—Neuralgia of the eyebrows and temples, pain passing over the frontal region and the eye of the same side, which is reddened and full of tears. Pain is hammering, burning, stitching, or tearing. Slightly relieved by pressure with the hand. Aggravations from light, fresh air, or any motion of the head. The paroxysms are periodical and begin with yawning and chilliness.—*The Medical Visitor*, April, 1895.

